# Context-specific adaptation for head fakes in basketball: a study on player-specific fake-frequency schedules

**DOI:** 10.1007/s00426-024-01977-2

**Published:** 2024-05-28

**Authors:** Iris Güldenpenning, Nils T. Böer, Wilfried Kunde, Carina G. Giesen, Klaus Rothermund, Matthias Weigelt

**Affiliations:** 1 Department of Sport & Health, University of Paderborn, Warburger Str. 100, 33098 Paderborn, Germany; 2 Department of Psychology, Würzburg University, Röntgenring 11, 97070 Würzburg, Germany; 3 General Psychology, Health & Medical University, Am Anger 66-73, 99084 Erfurt, Germany; 4 Department of General Psychology II, Friedrich Schiller University Jena, Am Steiger 3/Haus 1, 07743 Jena, Germany

## Abstract

In basketball, an attacking player often plays a pass to one side while looking to the other side. This head fake provokes a conflict in the observing opponent, as the processing of the head orientation interferes with the processing of the pass direction. Accordingly, responses to passes with head fakes are slower and result in more errors than responses to passes without head fakes (head-fake effect). The head-fake effect and structurally similar interference effects (e.g., Stroop effect) are modulated by the frequency of conflicting trials. Previous studies mostly applied a block-wise manipulation of proportion congruency. However, in basketball (and also in other team sports), where different individual opponents can be encountered, it might be important to take the individual frequency (e.g., 20% vs. 80%) of these opponents into account. Therefore, the present study investigates the possibility to quickly (i.e., on a trial-by-trial basis) reconfigure the response behavior to different proportions of incongruent trials, which are contingent on different basketball players. Results point out that participants indeed adapted to the fake-frequency of different basketball players, which could be the result of strategic adaptation processes. Multi-level analyses, however, indicate that a substantial portion of the player-specific adaptation to fake frequencies is accounted by episodic retrieval processes, suggesting that item-specific proportion congruency effects can be explained in terms of stimulus-response binding and retrieval: The head orientation (e.g., to the right) of a current stimulus retrieves the last episode with the same head orientation including the response that was part of this last episode. Thus, from a theoretical perspective, an attacking player would provoke the strongest detrimental effect on an opponent if s/he repeats the same head movement but changes the direction of the pass. Whether it is at all possible to strategically apply this recommendation in practice needs still to be answered.

## Introduction

In basketball games, players often perform a head fake: They play a pass in one direction (e.g., to the left) while orienting the head and gaze into the other direction (i.e., to the right). Because head orientation conflicts with pass direction, reacting to a pass with a head fake is slower and results in more errors than reacting to a pass without a head fake (e.g., Güldenpenning, Kunde. et al. 2020; Güldenpenning, Schütz Güldenpenning et al., 2020a, b; Kunde et al., 2011; Weigelt et al., 2017). This so-called head-fake effect reflects an interference effect, which can also be observed in other conflict tasks (i.e., Stroop task, Eriksen flanker task, Simon task). Interference effects occur as participants process the task-irrelevant stimulus feature incidentally. However, previous studies showed that the strength of the interference effect differs between experimental blocks that vary in the proportion of congruent and incongruent trials. Interference effects are larger in blocks where incongruent trials are rare (e.g., 20% of the trials) and smaller in blocks where incongruent trials are frequent (e.g., 80% of the trials; list-wide proportion congruency effect, LWPCE; Bugg, 2017). Such a frequency-based modulation of the interference effect has also been observed for the head fake in basketball (Alhaj Ahmad Alaboud et al., 2012; Güldenpenning et al., 2018). Laboratory studies have shown that the reaction time and the head-fake effect increases when reacting to video sequences with a whole-body movement as if to intercept the perceived pass (Alhaj Ahmad Alaboud et al., 2016; Güldenpenning et al., 2020a; Güldenpenning, Schütz Güldenpenning et al., 2020a, b). Nevertheless, it is still unclear to what extent previous studies and the results of the present study can be transferred to far more complex settings in sport.

For some time, researchers concordantly suggested that the LWPCE reflects attentional control, as the cognitive system varies attention weight between the task-relevant and the task-irrelevant stimulus feature in dependence of proportion congruency (Gratton et al., 1992; Logan & Zbrodoff, 1979; Lowe & Mitterer, 1982). This strategy is also termed a global attention strategy because it integrates knowledge about probabilities (of congruent and incongruent trials) that are based on multiple trials, and – once established – is used for an entire experiment or at least for a block in an experiment. To date, however, it is under debate if the LWPCE really reflects a global strategic process or rather a stimulus-driven control mechanism (Braem et al., 2019; Bugg & Crump, 2012; Rothermund et al., 2022). The former idea that proportion congruency effects in Stroop tasks are the result of experiment-wide attentional strategies was initially challenged by Jacoby et al. (2003). Jacoby and colleagues (2003) manipulated proportion congruency at the level of individual items in a Stroop task. A set of words (e.g., the words red, yellow, and white) was assigned to a mostly congruent (MC) condition, whereas another set of words (e.g., black, blue, and green) was assigned to a mostly incongruent (MI) condition. MC items were presented in their congruent color in 80% of the trials and in an incongruent color in 20% of the trials. For the MI items, these rates were reversed. The complete set of items was randomly intermixed. Regarding the list-wide proportion congruency, this design revealed a 50/50 ratio of congruent/incongruent trials. Accordingly, participants were not able to predict whether the subsequent item would be a congruent or incongruent one and they thus could not apply a global attention strategy (e.g., Gratton et al., 1992). Nevertheless, participants showed a proportion congruency effect, that is, a higher interference effect for MC items and a smaller interference effect for MI items. Jacoby et al. (2003) concluded that this item-specific proportion congruency effect (ISPCE) reflects a stimulus-driven retrieval of a stimulus attention association, which in principle could also be responsible for the proportion congruency effect found for list-wide manipulations (see also Braem et al., 2019). Specifically, MI items and MC items appear to be associated with different attention filters. If an item appears on the screen, the corresponding attention filter is automatically retrieved and provides control over the processing of the item. Thus, attention strategies – associated with a specific item – might reflexively be triggered by the appearance of that item and allow online control over the processing of potentially interfering stimulus features. In other words, the weight that is given to processing task-irrelevant information is not determined prior to the stimulus, but only while the stimulus is processed (for similar findings, see Bugg & Hutchison, 2013; Bugg et al., 2011).

Relatedly, further studies showed that attention strategies might also be bound to a task-irrelevant context in which the item occurs (so-called context-specific proportion congruency effect, CSPCE; e.g., Crump et al., 2006; Heinemann et al., 2009; Schouppe et al., 2014). Crump et al. (2006) applied a prime-probe version of a Stroop task. After a centrally presented color-word-prime (RED, GREEN, BLUE, YELLOW), a shape (circle, square) appeared below, or above fixation and participants had to name the color of the shape. The location was fixed for each shape (i.e., a circle always appeared below and a square always appeared above, or vice versa). One shape-location context was mostly congruent (i.e., the relation between word prime and shape color), whereas the other shape-location context was mostly incongruent. Comparable with the study of Jacoby et al. (2003) (and also with the LWPCE; Bugg, 2017), the Stroop effect was larger in the context of a high proportion of congruent trials and smaller in the context of a high proportion of incongruent trials (Experiment 1). Crump et al. (2006) therefore concluded that also task-irrelevant contextual information (e.g., location) can cue the retrieval of attentional control processes, as described by Jacoby et al. (2003). Other task-irrelevant contextual features, which can retrieve control processes, are color (Lehle & Hübner, 2008), the shape surrounding the stimulus (Schouppe et al., 2014), the temporal presentation windows (Wendt & Kiesel, 2011), the format of alphanumerical stimuli (Reuss et al., 2014), or human faces (Cañadas et al., 2013; Hutcheon, 2022).

While it is now undisputed that conflict adaptation can occur reflexively and at a trial-by-trial level, there is growing doubt that these adjustments have anything to do with attention or cognitive control (Rothermund et al., 2022; Schmidt & Lemercier, 2019). Rather, different kinds of proportion congruency effects can also be explained with alternative theoretical accounts. One popular explanation refers to contingency learning (Schmidt et al., 2007; Schmidt & Besner, 2008). Participants learn contingencies between a task-irrelevant stimulus feature (e.g., word meaning) and a response, and use these contingencies to predict the response associated with the distracting word (for variations in contingency learning processes, see Hutcheon, 2022). Besides contingency learning, episodic stimulus-response retrieval processes might also explain proportion congruency effects (for an overview, see Frings et al., 2020). This account claims that the distractor feature (e.g., word meaning) of a current stimulus (e.g., color word) retrieves the most recent episode in which the stimulus has been presented before (so-called “law of recency”; Giesen et al., 2020). By activating this last episode, the response that was executed during this previous episode is retrieved. Exemplarily for a Stroop-task, this means that for words presented mostly in congruent conditions, the probability is high that these words were presented in the same color also in the previous trial in which they occurred. For words presented mostly in incongruent conditions, the probability of word-color repetitions, however, is low. Thus, within mostly congruent conditions, there is a high proportion of trials in which the retrieval of the response stored with the word from the previous episode facilitates the current response. Within mostly incongruent conditions, on the other hand, there is only a low proportion of trials in which the retrieval of the response stored with the word from the previous episode facilitates the current response. At the same time, for the mostly congruent items, there is a lower proportion of trials in which the word meaning of the previous episode activates the incorrect response, and for the mostly incongruent items, there is a higher proportion of trials in which the word meaning of the previous episode activates the incorrect response. Thus, adaptations to the proportion of incongruent trials can be explained by the proportion of repetition/change of responses tied to the distractor of a previous episode (e.g., Giesen et al., 2020; Schmidt et al., 2020).

Taken together, like contingency learning, episodic retrieval is based on a form of stimulus-response association, but unlike contingency learning, it depends solely on the most recent episode in which the current stimulus previously occurred, and not on a trial-wise learning and application of frequent stimulus-response contingencies. Recent studies showed that episodic retrieval of the most recent episode is a better predictor for performance than contingency learning; what is more, it may also explain effects of stimulus-response contingencies to a large extent (Giesen et al., 2020; Schmidt et al., 2020; Rudolph & Rothermund, 2024; Xu & Mordkoff, 2020). Together, both cognitive control accounts (Jacoby et al., 2003) and accounts, which propose some kind of stimulus-response associative learning (Frings et al., 2020; Giesen et al., 2020; Hutcheon, 2022; Schmidt & Besner, 2008), can explain the adaptation to proportion congruency at a trial-by-trial level.

Experimentally dissociating the different theoretical approaches from each other is difficult (i.e., different accounts make similar predictions), in some cases not possible, and sometimes results in very artificial experimental arrangements far away from the actual proportion congruency situations (for a more detailed discussion, see Rothermund et al., 2022). The focus of the present study is to investigate trial-by-trial adaptations to proportion congruency in an experimental setting, which mimics a one-on-one player situation in basketball games. Here (and also in other games sports), context-specific adaptation is of critical interest, for example, if a defending basketball player needs to spontaneously adapt to the individual frequency of head fakes of different opponents. One opponent player might be known to fake very often (e.g., in 80% of all one-on-one situations), whereas another opponent player might be known to fake only sparsely (e.g., in 20% of all one-on-one situations). These different players get incidentally into one-on-one situations with the defending player, and it might be a benefit for the defender if their behavior would rapidly adjust to match the fake frequency of the attacking player. Therefore, the present study investigates whether participants adapt to player-specific fake-frequency schedules. Furthermore, we investigated whether such adaptations to player-specific fake frequencies can be explained by episodic retrieval of the last encounter with the respective player and the previously executed response (cf. Rothermund et al., 2022).

**Fig. 1:**
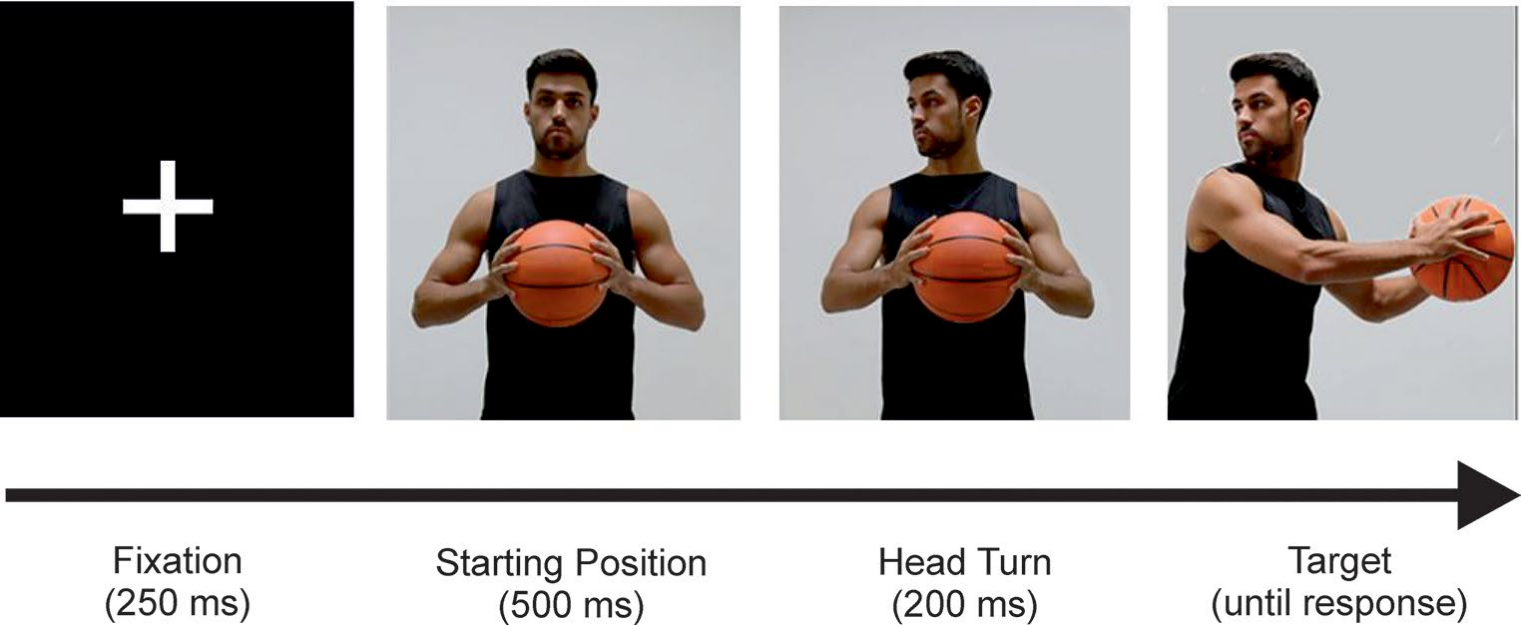
Stimulus material used in the study, exemplarily for a pass with head fake to the right side

To this end, we conducted a reaction time experiment with three basketball players as target stimuli. Before the experiment, participants were informed about the different fake-frequency schedules (i.e., 20%, 50%, 80%) of these players, which were then randomly presented during the experiment. Rather extreme proportions of head fakes (20%, 80%) were chosen in this study to have strong manipulations and to unequivocally distinguish between different conditions and their underlying processes. In line with studies on episodic retrieval processes mentioned above (e.g., Giesen et al., 2020; Schmidt et al., 2020; Rudolph & Rothermund, 2024; Xu & Mordkoff, 2020), but contrary to the dynamic information in real sports games, we used photographic stimulus material (cf. Figure 1). With photographic material, it is possible to control the features of the three different basketball players perfectly. With video material, it is virtually impossible to create recordings of passes with and without head fake that are similar in all relevant respects, as each player performs the head fake with slight variations. For reasons of standardization, we have therefore used photographic stimuli. We predicted that participants adapt to different player-specific fake-frequency schedules and that the head-fake effect is more pronounced for the basketball player, who fakes sparsely (i.e., 20%), than for the basketball player, who fakes very often (i.e., 80%). Following the standard variance analysis procedures, we post-hoc analyzed the data with a multilevel modelling technique (Rothermund et al., 2022, see also Giesen et al., 2020) that allows us to investigate whether the observed adaptation to player-specific fake frequencies can be explained by episodic retrieval of the last, most recent episode by taking into account whether the response executed in the previous trial would match (vs. mismatch) with the currently required response.

## Methods

### Participants

Planning of the sample size was carried out using MorePower 6.0.4 (Campbell & Thompson, 2012). Previous studies, which investigated frequency-based modulations of the head-fake effect, reached large effect sizes (ɳ_p_^2^ > 0.14) for the interaction effect between *type of pass* and *fake frequency* (Alhaj Ahmad Alaboud et al., 2012; Güldenpenning et al., 2018). Accordingly, a large effect size (ɳ_p_^2^ = 0.14/*f* = 0.40), with power set at 0.8 for a 2 × 3 within-participants factorial design, with the factors *type of pass* (genuine pass vs. deceptive pass) and *fake frequency* (20%, 50%, 80%), yielded a recommended total sample size of 32 for the interaction effect. Thirty-five participants were tested. Data from one participant was excluded due to an error in the programming of the experiment.

Data of thirty-four students from Paderborn University were analysed for the present study (13 females, 21 males; mean age = 22.0; *SD* = 2.7). All participants studied sport science and had basic sporting skills, but no specific experience in basketball. All participants reported normal or corrected-to-normal vision and had no knowledge of the expected outcome of this experiment. Each participant gave informed consent to participate. They were not paid for participation. All rights of the participants were protected, and all experiments were carried out according to the sixth revision (Seoul, 2008) of the 1964 Declaration of Helsinki.

### Apparatus and stimulus material

Twenty-one photographic pictures of two male basketball players and one female basketball player were used in the present study. All basketball players wore identical black basketball shirts and black shorts during stimulus recording. Pictures were taken while the players imitated to pass the ball to the left or to the right side, either with or without performing a head fake (for an example of the stimulus material, see Fig. 1). For each of the basketball players, one picture displayed the starting position in which the player held the ball in front of the body at chest height. At two further pictures, the basketball players were displayed with their head rotated either to the left side or to the ride side, but still having the ball in front of the chest. Four other pictures of each basketball player were the target pictures of the study. At the targets, the basketball player imitated a pass to one side (i.e., either to the left side or to the right side) whilst the head was rotated either to the same side (i.e., pass without head fake, hereafter referred to as genuine pass) or to the opposite side (i.e., pass with head fake, hereafter referred to as deceptive pass). The size of the stimuli was 900 × 1200 pixel with a resolution of 72dpi. All stimuli were presented in color on a black background of a 24-inch TFT-monitor. The presentation of the stimulus material was controlled with an IBM-compatible personal computer and the software Presentation (Version 14.5, http://www.neurobs.com). Responses to the targets were single key presses on a standard computer keyboard and were carried out with the index fingers of each hand, with the “Alt”-key (for genuine and deceptive passes to the left side) and the “Alt Gr”-key (for genuine and deceptive passes to the right side).

### Procedure and Design

Participants were given written instructions. They were informed that three different basketball players will occur during the experiment^1^. These players would either be shown while passing the ball to the left/right side, performing a genuine or a deceptive pass (factor *type of pass*). The *fake frequency* (20%, 50%, 80%) depended on the player. Participants were specifically informed about the fake frequency of each player. This information was repeated between each experimental block (see below). The fake frequency of the different stimulus models was counterbalanced between participants. Participants were asked to respond as fast and as accurately to the pass direction shown at the target picture of the stimulus sequence. The “Alt” - button had to be pressed with the left index finger when a pass (with or without head fake) was played to the left side and the “AltGr” - button had to be pressed when a pass (with or without head fake) was played to the right side. The first block of 12 trials was considered as practice to familiarize participants with the experiment. Data from this block were not analyzed. This practice block was followed by three test blocks of 120 trials each (resulting in a total of 360 trials), which were separated by short breaks if participants wanted to rest.

Each trial began with the presentation of a central fixation cross (250 ms). Afterwards, the sequence of three pictures of one of the three basketball players was shown (cf. Figure 1): the starting position (500ms), the head rotation (200ms), and the target with the player performing a genuine or a deceptive pass either to the left or right side. A sequence of images was used, which is comparable with previous studies (e.g., Hutcheson & Spieler, 2017; Crump et al., 2016). We set the interstimulus interval (ISI) between the third and second image to 200ms, as this time interval very reliably causes a head-fake effect (Polzien et al., 2021) and is also comparable to the time offset that we find in video sequences of a faking basketball player (Güldenpenning et al., 2020). The target remained on the screen until a response was given. After the trial ended, participants received feedback about their answer. If there was an error, the word “Fehler” (German for “error”) appeared on the screen for 500 ms, followed by a 500 ms blank screen. If they responded correctly a blank screen would be displayed for 1000ms.

### Data analysis

We analysed the mean reaction times (RT) of the correct responses and the proportion of incorrect responses (= error rate, ER; 2.9%). Also, 0.3% of the data was excluded from further analyses, because RTs were either below 100 ms (considered as anticipations) or higher than 1000 ms (considered as outliers). Mean RTs and mean ERs were submitted to an ANOVA with the factors *type of pass* (genuine pass vs. deceptive pass), and *fake frequency* (20%, 50%, 80%) as repeated measures. Post hoc *t*-tests were corrected according to Holm-Bonferroni (Holm, 1979). A violation of the sphericity-assumption resulted in a correction of the *p*-values according to Greenhouse-Geisser. However, due to better readability, we report the uncorrected degrees of freedom^2^. The post-hoc multilevel modelling technique is reported and explained after the results of the ANOVA (section “Episodic retrieval of responses”).

## Results

### Reaction times

**Fig. 2:**
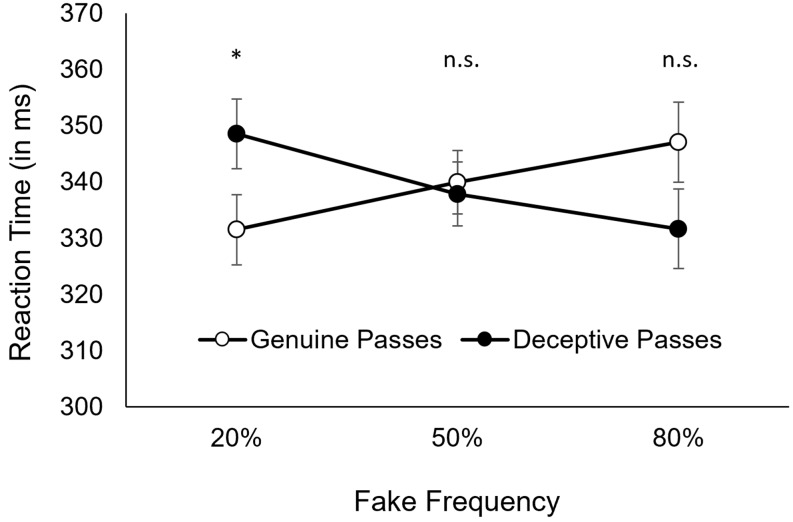
Reaction times for genuine and deceptive passes, as a function of fake frequency. *Note* Reaction times for genuine passes (unfilled circles) and deceptive passes (filled circles), as a function of fake frequency. Error bars represent the 95% confidence interval of the difference between genuine und deceptive passes (i.e., confidence interval for paired differences = CI_PD_; cf. Pfister & Janczyk 2013). Two means from paired samples are significantly different if one mean is not included in the CI_PD_ around the other mean. Notably, for 80% fake frequency, the mean is not included in the CI_PD_, however, significance testing is based on corrected p-values here

Reaction times are illustrated in Fig. 2. The analysis of RTs revealed neither a main effect for *type of pass, F*(1, 33) = 0.00, *p* = .972, ɳ_p_^2^= 0.00, nor for *fake frequency, F*(2, 66) = 0.16, *p* = .855, ɳ_p_^2^= 0.01. However, the interaction between *type of pass* and *fake frequency* was significant, *F*(2, 66) = 7.47, *p* = .003, ɳ_p_^2^= 0.19, *ε* = 0.82. For 20% fake frequency, participants reacted significantly faster to genuine passes (*M* = 332ms; 95% CI [*318, 345*]) than to deceptive passes (*M* = 349ms, 95% CI [*337, 361*]), *t*(33) = 2.79, *p* = .027, *d*_z_= 0.47. For 50% fake frequency, participants reacted equally fast to genuine passes (*M* = 340ms, 95% CI [*326, 354*]) and to deceptive passes (*M* = 338ms, 95% CI [*326, 349*]), *t*(33) = 0.38, *p* = .710. For 80% fake frequency, participants reacted considerably slower to genuine passes (*M* = 347ms, 95% CI [*333, 361*]) than to deceptive passes (*M* = 332ms, 95% CI [*320, 344*]), however, the corrected *p*-value for this “reversed” head-fake effect was not statistically significant, *t*(33) = 2.21, *p* = .068.

### Error rates

**Fig. 3:**
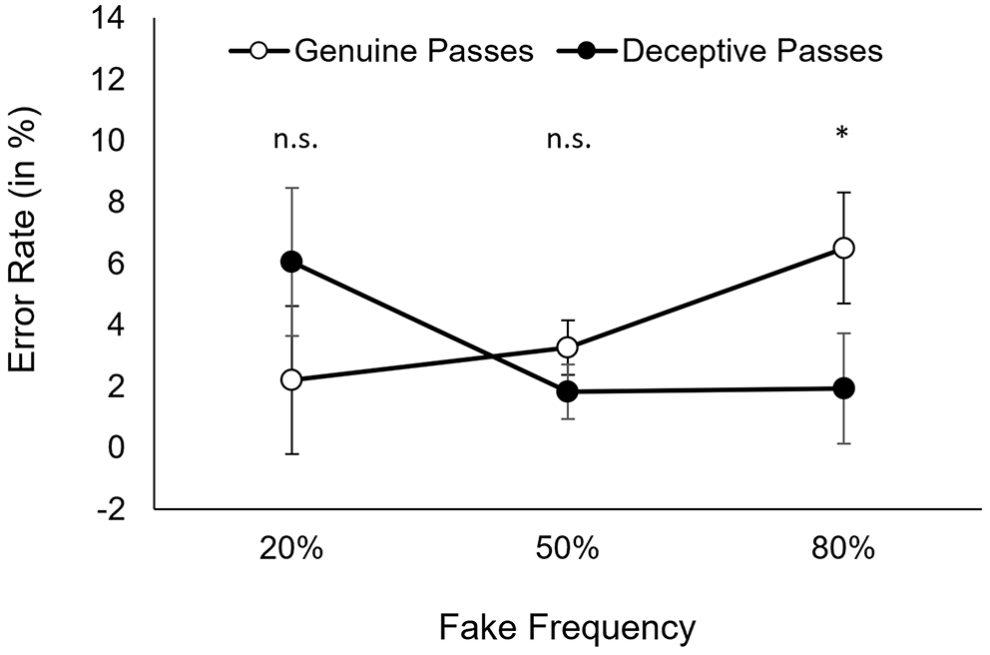
Error rates for genuine and deceptive passes, as a function of fake frequency. *Note* Error rates for genuine passes (unfilled circles) and deceptive passes (filled circles), as a function of fake frequency. Error bars represent the 95% confidence interval of the difference between genuine and deceptive passes (i.e., confidence interval for paired differences = CI_PD_; cf. Pfister & Janczyk 2013). Two means from paired samples are significantly different if one mean is not included in the CI_PD_ around the other mean

Error rates are illustrated in Fig. 3. The analysis of ERs revealed neither a significant main effect for *type of pass, F*(1, 33) = 0.67, *p* = .419, ɳ_p_^2^= 0.02, nor for *fake frequency, F*(2, 66) = 1.77, *p* = .178, ɳ_p_^2^= 0.05, *ε* = 0.86, however, the interaction between *type of pass* and *fake frequency* was significant, *F*(2, 66) = 5.07, *p* = .024, ɳ_p_^2^= 0.13, *ε* = 0.60. For 20% fake frequency, participants performed less errors to genuine passes (*M* = 2.2%; 95% CI [*0.9, 3.5*]) than to deceptive passes (*M* = 6.1%, 95% CI [*1.2, 10.9*]), but this effect was not significant, *t*(33) = 1.62, *p* = .212. For 50% fake frequency, reactions to genuine passes were slightly, but not significantly (*p* > .05) more error prone (*M* = 3.3%, 95% CI [*1.8, 4.8*]) than to deceptive passes (*M* = 1.8%, 95% CI [*1.1, 2.9*]), *t*(33) = 1.66, *p* = .212. For 80% fake frequency, participants performed significantly more errors to genuine passes (*M* = 6.5%, 95% CI [*3.0, 10.1*]) than to deceptive passes (*M* = 2.0%, 95% CI [*1.1, 2.8*]), *t*(33) = 2.59, *p* = .042, *d*_z_= 0.45.

### Episodic retrieval of responses

We investigated whether stimulus-specific response retrieval effects influenced task performance and whether this can explain the adaptation to player-specific head fake frequencies. RT data were analyzed with a linear mixed-effects model (LMM), using trials as units of analysis. Analyses were performed with SPSS (version 28, MIXED command). Participants were treated as level-2 predictor (i.e., trials were nested within participants) to account for statistical dependence between trial-based performance. In detail, we computed a random intercept model and included trial RT as dependent variable. In Model 1, we added fixed effects for *type of pass* (contrast coded with *genuine passes* = 0.5, *deceptive passes*=-0.5) and *fake frequency* (20% fakes = 0.5, 80% fakes=-0.5; players with equal amount of genuine and fake passes were excluded from this analysis) and their interaction. Last, we included participants as a random effect. Trials with erroneous responses were excluded from the RT analyses. To ensure that the analysis without the episodic retrieval factor was based on exactly the same data as the analysis including episodic retrieval, trials in which an incorrect response had been given during the last occurrence of the player, and trials in which the player had been shown with a different head movement during the last occurrence were also excluded (for details see below). Analogously to the ANOVA results, Model 1 yielded only a significant interaction between *type of pass* and *fake frequency*, *b*=-25.44, *t*(3632.516) = -5.40, *p* < .001, both main effects were non-significant (*type of pass*: *b* = 1.19, *t*[3632.674] = 0.51, *p* = .61; *fake frequency*: *b* = 1.67, *t*[3632.545] = 0.71, *p* = .48).

To test whether this effect can be explained by stimulus (here: player)-specific episodic retrieval of matching (vs. mismatching) previous responses, we entered *response match* between the current trial and the last occurrence of the same stimulus (i.e., player and head orientation) as an additional predictor to the LMM analyses in Model 2. Trials in which the player of the current trial had been presented with a different head movement during its last occurrence were also eliminated, since it is unclear which episode would be retrieved in those trials: The temporally closer episode with a partial match (same player, different head movement) or the more distant episode with a full match (same player, same head movement). Because the LMM analysis requires centering of predictors and more response matches (approx. 67%) than response mismatches (approx. 33%) occurred in the dataset, the previous response match predictor was contrast coded with *previous response matches* = 0.33 and *previous response mismatches*=-0.67, respectively. Model 2 yielded a significant main effect of previous response match, *b*=-12.77, *t*(3631.128) = -5.48, *p* < .001, reflecting faster performance if the response of the current trial matched the response that was executed on the last previous occurrence of the same stimulus (here: head orientation of a particular player). Importantly, the interaction of *type of pass* and *fake frequency* was no longer significant after entering the response match factor into the model, *b*=-9.57, *t*(3631.327) = -1.74, *p* = .08. This implies that episodic response retrieval accounts for a substantial amount of variance in the head-fake effect and even renders the effect non-significant for the RT data. No other effect was significant (*type of pass*: *b* = 1.30, *t*[3631.673] = 0.56, *p* = .58; *fake frequency*: *b* = 1.54, *t*[3631.542] = 0.66, *p* = .51).

The same multi-level analyses were also conducted for accuracy data, coding correct responses as 1 and errors as 0. In Model 1 (without the response match factor), the t*ype of pass* × *fake frequency* interaction was significant, *b* = 0.084, *t*(3757.473) = 6.26, *p* < .001, replicating the interaction that was obtained in the ANOVA analyses of the error data. Including *response match* (response match = 0.33, response mismatch = 0.67) as an additional predictor in Model 2 yielded a significant effect for this predictor, *b* = 0.023, *t*(3756.647) = 3.48, *p* < .001, indicating that less errors were made if the same response was required in the current and previous trial. Deviating from the RT analyses, however, inclusion of the *response match* factor did not completely eliminate the *type of pass* × *fake frequency* interaction, *b* = 0.054, *t*(3757.772) = 3.43, *p* < .001, which was still significant although strongly reduced in size (the effect of the interaction was reduced by 36% after controlling for effects of episodic retrieval).

## Discussion

In basketball games, it is important to adapt to the individual fake frequency (e.g., 20% vs. 80%) of different opponents once one of these individual opponents is encountered. The mechanisms underlying such adaptations could be either strategic (i.e., contingency learning) or rather be based on random repetition/change of stimulus-response associations (i.e,. episodic response retrieval processes). The present study therefore investigates the cognitive mechanisms when participants quickly (i.e., on a trial-by-trial level) reconfigure their response behavior to player-specific fake-frequency schedules. Stimulus material of three different basketball players was used, and each player was presented with a different frequency of head-fake trials (i.e., 20%, 50%, 80%). This manipulation is comparable with studies designed to investigate the context-specific proportion congruency effect (CSPCE; e.g., Hutcheon, 2022). In this study, proportion congruency was contingent on the player, who determines the specific stimulus or item. The analysis of the mean RTs revealed a frequency-based modulation of the head-fake effect, that is, the head-fake effect was present for 20% head fakes and disappeared for 50% head fakes. Also, the head-fake effect reversed (though not significantly) for 80% head fakes. This pattern of results is generally supported by the mean ERs, that is, the head-fake effect occurred clearly on a descriptive level for 20% head fakes, and the reversed effect was significant for 80% head fakes. Note, however, that the strongest test for the player-specific adaptation to fake frequencies is the interaction between type of pass and fake frequency, which was significant for both, RT and ER.

Results thus indicate that participants adapted to the individual frequency of head-fake usage of the basketball player displayed in a trial. The pattern of results found here varies from the standard finding of proportion congruency effects, namely of a decreasing interference effect with a decreasing proportion of congruent trials (Alhaj Ahmad Alaboud et al., 2012; Güldenpenning et al., 2018; Gratton et al., 1992; Lowe & Mitterer, 1982). Instead, the head-fake effect was evident for 20% head fakes, but already disappeared for 50% head fakes, and even reversed (although not significant in RTs) for 80% head fakes. This pattern of findings is comparable with data reported by Jacoby et al. (2003), who observed higher interference effects for items that were presented mostly in congruent word-color combinations (MC items) than for items that were presented mostly in incongruent word-color combinations (MI items). According to Jacoby et al. (2003), the ISPCE is due to stimulus-specific retrieval of attentional filters – that is, attentional strategies that affect to which degree (ir)relevant features of a stimulus/item are processed (or not). If an item appears on the screen, the corresponding attention filter is automatically retrieved and provides control over the processing of the item. Thus, attention strategies – associated with a specific item – might be triggered by the appearance of that item and allow online control over the processing of potentially interfering stimulus features. In other words, the weight that is given to processing task-irrelevant information is not determined prior to the stimulus, but only while the stimulus is processed (i.e. mostly congruent or mostly incongruent) (for similar findings, see Bugg & Hutchison, 2013; Bugg et al., 2011). Although, such an account can explain weaker congruency effects for items with a high proportion of incongruent trials, or even the absence of a congruency effects if the irrelevant stimulus dimension is completely blocked from processing, it cannot explain the reversal of the congruency effect for the mostly incongruent (MI) items (see Klauer et al., 2003, for a similar argument).

A theoretical account that can explain the reverse effect is contingency learning (Schmidt et al., 2007; Schmidt & Besner, 2008). In our study, participants might have learned associations between the context (e.g., the type of player) and the head orientation. Specifically, when the head is oriented to the right for a basketball player, who fakes in 80% of the trials, the correct response is typically left. If, however, the head is oriented to the right for a basketball player who fakes only in 20% of the trials, the correct response is typically right. When participants use these associations between type of player and head orientation, this results in very fast responses to passes with head fakes for the basketball player, who fakes in 80% of the trials, and to passes without head fakes for the basketball player, who fakes in 20% of the trials. In contrast, responses are slow to passes without head fakes for the basketball player, who fakes in 80% of the trials, and to passes with head fakes for the basketball player, who fakes in 20% of the trials (for variations in the contingency learning process, see Hutcheon, 2022). When participants rely *completely* on the compounded information of context and head orientation, the head-fake effect reverses for the basketball player who fakes in 80% of the trials.

Beyond contingency learning CSPC might also be explained by episodic stimulus-response retrieval processes (for an overview, see Frings et al., 2020). Accordingly, we investigated whether stimulus-specific adaptation to fake frequencies can be explained by stimulus-driven episodic retrieval of previous responses that were executed at the last occurrence of the stimulus. This idea was motivated by recent findings from Rothermund et al. (2022), who could show that block-wise proportion congruency effects can be completely eliminated (and thereby: explained) by statistically controlling for episodic retrieval of incidental stimulus-response episodes. Such an account can also explain the reversal of the head-fake effect for players, who show a high frequency of incongruent head orientations: For these players, episodic retrieval has a high chance of retrieving a matching response from the last episode in which the same player showed the same head movement, since this last episode would also be an incongruent trial in 80% of the cases. Conversely, episodic retrieval would lead to interference on congruent trials for these players because a mismatching response would be retrieved with a high likelihood from the last occurrence of the specific combination of player and head orientation, since 80% of trials from the last episode would be an incongruent trial.

The findings from a sophisticated multilevel modelling technique are interesting: For the RT data, the interaction between type of pass and fake frequency (which is indicative for player-specific adaptation of the head-fake effect) was no longer significant as we statistically controlled for stimulus-driven episodic retrieval effects. This means that if we entered *response match* between the current trial and the last occurrence of the same stimulus (i.e., player and head orientation) as an additional predictor to the LMM analyses, the interaction between type of pass and fake frequency could be explained by stimulus (here: player)-specific episodic retrieval of matching (vs. mismatching) previous responses. Accordingly, episodic response retrieval accounts for a substantial amount of variance in the head-fake effect. For the accuracy data, the interaction was not completely eliminated, but still significant, which might indicate that error data reflect more strategic responding that might be based on abstract contingency awareness.

Furthermore, there are reasons why episodic retrieval processes might have been less strong compared to previous studies. Most importantly, in the current study participants were explicitly informed about player-specific fake frequencies before the start of the study. Recent data indicated that stimulus-driven episodic retrieval processes explain less variance in contingency learning effects as soon as participants are aware of the underlying contingencies (for instance, because they detect these contingencies themselves: Arunkumar et al., 2022; or because they are explicitly instructed about them: Giesen et al., 2024; Rudolph et al., 2024). Awareness or knowledge about player-specific fake frequencies therefore represents a different process to decide on how to act. Importantly, knowledge is not based on episodic retrieval processes, but rather relies on applying detected (or instructed) rules (here: fake frequencies per player). Adding to these strategic effects, the trial sequence itself that was used in the present study might have favored strategic responding in the current study, because we used a sequence of three images in this study (see Fig. 1). The first image showed the player in a neutral position. This starting position appeared 700 ms before the target, which provided ample time to strategically prepare the response contingency based solely on the (instructed) knowledge of player-specific fake frequencies.

Taken together, our data demonstrate a strong influence of episodic retrieval processes in explaining context-specific congruency effects. Suggesting that episodic retrieval is a fundamental process of action control, context-specific adaptation can also be expected to be present in different sport settings. Accordingly, the current study demonstrates the importance of considering the “law of recency” (Giesen et al., 2020) as an important explanatory mechanism for real-life adaptation effects in the domain of sports. The fact that the most recent episode has a strong influence on current behavior would imply that repeating the same head movement but changing the direction of the pass (i.e., faking with the head movement to pass to the same side as before, but actually passing to the contrary side) should have the strongest misleading effect on the opponent – of course, given that it is the same player you are trying to mislead.

It is questionable whether it is at all possible to strategically carry out the sequence of head-fakes according to this theory-based recommendation, as other factors also determine the best possible fake action, such as your own position on the field and that of your team-mates and opponents. Thus, even if we think that episodic retrieval processes also play an important role for head fake scenarios, other situations in sport may be better suited to strategically utilize this process. Think of the penalty situation in handball, where the attacker can fake a throw several times before actually throwing. The attacker could look to the left corner of the goal and fake a throw there. The subsequent actual throw, however, goes to the right while the gaze direction remains to the left. As the gaze direction of the actual throw retrieves the response of the fake throw before, the gaze direction would retrieve the “wrong” response in the goalkeeper.

The results of the present study raise the general question of whether coaches should provide athletes with information about action preferences of their opponents. Both strategic (i.e., contingency learning) and automatic adaptation processes (i.e., episodic retrieval) seem to lead to similarly efficient behavior. However, we suggest that episodic retrieval processes, which take part without explicit information, are superior to strategic processes in a real sports context, as they do not cause further cognitive load. It seems worthwhile to investigate whether explicit instructions might even disturb automatic context adaptation processes in dynamic sport settings.

## Footnotes

1 In a previous study (Güldenpenning et al., 2023), we manipulated the probability that a pass is played to the left or right side (i.e., 25%, 50%, 75%, respectively) and compared the head-fake effect between two groups: One group received explicit information (i.e., instructions) about the action preferences, whereas the other group did not. Action probability impacted processing of the head fake independent of previous instructions. Probability information, thus, is recognized even without prior instruction and taken into account during processing.

2 A violation of the sphericity-assumption requires modifications of the degrees of freedom (df’s) to reduce the Type I error. The estimates for this modification are denoted by epsilon (ε), providing a measure of deviation from sphericity. An epsilon of 1 indicates that sphericity is exactly met, smaller epsilon values indicate increasing deviation. Due to better reading, the uncorrected df’s are given for the F-value, but the corrected df’s can be easily calculated by multiplying the original df’s with ε. Note that the overall pattern of results was the same for both, corrected and noncorrected degrees of freedom.

## Declarations

### Open practices statement

The datasets generated during and/or analysed during the current study are available from the OSF repository. The study was not preregistered.
